# Knowledge and skills required to perform point-of-care ultrasonography in family practice – a modified Delphi study among family physicians in Slovenia

**DOI:** 10.1186/s12875-020-01130-z

**Published:** 2020-03-26

**Authors:** Vesna Homar, Zala Kumse Gale, Mitja Lainscak, Igor Svab

**Affiliations:** 1 Department of Family Medicine, Faculty of Medicine, University of Ljubljana, Poljanski nasip 58, 1000 Ljubljana, Slovenia; 2 Faculty of Medicine, Department of Internal Medicine, University of Ljubljana, Ljubljana, Slovenia; 3 Center for Heart Failure, General Hospital Murska Sobota, Murska Sobota, Slovenia

**Keywords:** Point-of-care ultrasonography, Ultrasound, Family practice, Family medicine, General practice, Primary health care

## Abstract

**Background:**

More and more family physicians (FPs) are using point-of-care ultrasonography (POCUS) in Europe. Still, there is no general consensus about the specific knowledge and skills that a FP should acquire in order to effectively perform POCUS. The objective of this study was to identify indications for the use of POCUS among FPs, explore the barriers of its use and provide an expert opinion of FPs on knowledge and skills required to effectively implement POCUS in family practice.

**Methods:**

A modified two-round Delphi study was carried out among FPs using POCUS in Slovenia.

**Results:**

21 FPs were invited to participate in the study. A total of 13 FPs (62%) responded the round-one questionnaire and 10 (48%) completed the round-two questionnaire. Results show a large variability of indications for the use of POCUS in family practice, the most common being acute abdominal conditions, lung ultrasonography and eyeballing echocardiography. In contrast, the results show little variability in barriers for the use of POCUS, the most common being lack of time, inaccessibility of specific training programmes and financial issues. There is a strong consensus on the knowledge and skills needed to perform POCUS. Panellists agreed on a learning medical knowledge, technical skills and expressed a need for individual consultations and tutorship options.

**Conclusion:**

This study proves that although POCUS is used in family practice for a wide variety of indications with a significant number of barriers, there is a strong consensus on what a FP needs to know to effectively perform POCUS.

## Background

Point-of-care ultrasonography (POCUS) is a diagnostic tool increasingly used in various clinical specialties, including family medicine and general practice. It enables a clinician to visualise certain body areas and objectify clinical signs at patient’s bedside. Due to rapid technological advances, there are more compact, less expensive and technically improved ultrasound devices on the market [1]. It has a potential to become an important tool in family practice and may possibly reduce healthcare costs [2]. Studies showed that FPs performing POCUS demonstrate a high inter-rater agreement and reliability [3] and can gain important clinical information for further decision making using POCUS. Therefore, many family physicians (FPs) are considering implementing POCUS into their everyday practice [4, 5].

Although more and more FPs are using POCUS in Europe, there is no general consensus about the specific knowledge and skills that a FP should acquire in order to effectively perform POCUS. FPs using POCUS have obtained the knowledge and skills by following various teaching courses of organ-specific ultrasound or POCUS and they are pragmatically integrating their knowledge into everyday practice. Specific training standards have been developed for teaching POCUS to emergency and critical care physicians [6] and the training courses are being integrated in pre- and post graduate curricula [6–8]. American Association of Family Practice has published curriculum guidelines for teaching POCUS to residents of family medicine in 2018 [9]. However, there are no specific training standards for POCUS in family practice for already established specialists.

Besides the lack of specific training options in the use of ultrasound, there are also other barriers for its implementation in family practice. Previous studies have identified also financial aspects and time restrains [5, 10] as the other two most common limitations.

The aim of this study was to identify the indications for using POCUS among Slovenian FPs, explore the barriers for POCUS use among them and provide an expert consensus on knowledge and skills required to effectively implement POCUS in family practice.

## Methods

An expert two-round Delphi study was carried out among FPs using POCUS. The study was conducted in multiple primary healthcare centres in Slovenia from March to May 2016.

21 FPs, who had been using POCUS, were invited to participate in the expert panel of this study. The expert panel was formed of 13 experts who expressed an interest to participate in the study and who signed an informed consent. The panel consisted of six female and seven male physicians with an average of 14,8 years of practice. All FPs provided also home-visits and worked at least partly in the out-of-hours care or pre-hospital emergency service. All experts have completed at least one existing structured ultrasonography course.

Inclusion criteria were:

1. Being a family medicine or family practice specialist or trainee in Slovenia.
2. Using POCUS in family practice.

Experts have been self-evaluated as knowledgeable and skilled in the use of POCUS [11, 12]. There were no exclusion criteria regarding the formal education of POCUS, proficiency of using POCUS or the duration of using POCUS.

Participants of the expert panel were identified through purposive and snowball sampling methods. First, 21 personal invitation e-mails were sent to Slovenian FPs, who have been known for using POCUS in their practice. Next, FPs using POCUS have been requested to forward the invitation to other FPs using POCUS, who might have been unidentified by the conductors of the study.

The questionnaire used in your study was developed for this study and is available in the Additional file 1.

### Round-one questionnaire

The expert panel received a paper or electronic questionnaire with three open questions, each representing one outcome measure:

1. For which indications do you use POCUS in your family practice?
2. What are the barriers of use of POCUS in a family practice?
3. What knowledge and skills does a FP need to safely use POCUS in a family practice?

The answers were revised, coded and grouped by two independent reviewers to determine one list of outcomes for each outcome measure. The grouping was done by the same two reviewers and was not pre-defined. Any disagreements between reviewers were resolved by the team discussion of all four authors.”

### Round-two questionnaire

Panellists were requested to evaluate the frequency of use of POCUS for listed indications, the importance of listed barriers and the importance of listed knowledge and skills. A three-point Likert-like scale was used for outcome measures:

1. Indications for the use of POCUS: often (2) – sometimes (1) – never (0)
2. Barriers of the use of POCUS: very important (2) – important (1), not important (0)
3. Required knowledge and skills for the use POCUS: very important (2) – important (1), not important (0).

The survey was administered through a web-based survey tool (http://www.1ka.si) or in postal version, as preferred by the panellist.

## Results

13 panellists completed round-one questionnaire. Ten (77%) panellists completed the round-two questionnaire. All responses were complete and on topic.

### Round-one results

#### Indications

Panellists listed 34 different indications for the use of POCUS in family practice. The majority of panellists structured the indications according to organ systems. One panellist listed the indications with respect to various symptoms (e.g. abdominal pain, swelling of extremities etc.). One panellist did not list any indications, but openly discussed the advantages of use of POCUS in family practice.

34 indications were grouped in five categories according to organ systems: lungs, cardiovascular, abdominal, musculoskeletal and life-threatening situations.

#### Barriers

Panellists listed 13 barriers for the use of POCUS in family practice. All panellists mentioned more than one barrier, with time-issue being the most common.

13 barriers were grouped in 3 categories: organisation, education and finance.

#### Knowledge and skills

Panellists listed 11 different knowledge areas and skills that a FP should adopt for a safe use of POCUS. The structures of answers varied considerably. Some panellists focused on technical skills, some on background medical knowledge, some recommended established ultrasound courses or tutoring.

11 different knowledge and skills were grouped in 3 categories: knowledge, skills and education.

### Round-two results

#### Indications

The frequency of the use of POCUS in family practice for each indication is presented in Table 1. Number of panellists using POCUS for listed indications in family practice is given. Different scores were assigned for different frequencies: often (2 points), sometimes (1 point) or never (0 points) and total sum of assigned points was calculated; *n* = 10.

**Table 1:** The frequency of the use of POCUS in family practice

	Indications	Often (No. of panellists)	Sometimes (No. of panellists)	Never (No. of panellists)	Total score
Lungs	Pneumonia	3	5	2	11
	Pleural effusion	8	2	0	18
	Pneumothorax	8	2	0	18
	Pulmonary embolism	3	3	4	9
Cardiovascular	Heart size and motility	6	4	0	16
	Evaluation of hydration (vena cava compliance)	5	4	1	14
	Pericardial effusion and heart tamponade	6	4	0	16
	Abdominal aortic aneurism or dissection	7	3	0	17
	Abdominal aortic aneurism (screening)	8	1	1	17
	Deep venous thrombosis	8	2	0	18
Abdominal	Intra-abdominal free fluid	9	1	0	19
	Ileus	3	3	4	9
	Organomegaly	4	6	0	14
	Gallbladder disease, gallstones	9	1	0	19
	Hydronephrosis, renal stones	9	1	0	19
	Retention of urine	9	1	0	19
	Cystitis	2	0	8	4
	Evaluation of prostate	4	3	3	11
	Evaluation of testicles	1	5	4	7
	Pregnancy (confirmation, evaluation)	2	5	3	9
	Extra-uterine pregnancy	3	2	5	8
	Uterine myoma	1	2	7	4
	Ovarian cysts	1	2	7	4
Musculoskeletal	Injuries: muscle, tendon rupture, haematoma	4	5	1	13
	Bursitis	4	3	3	11
	Joint effusion	2	6	2	10
	Fractured bone	2	6	2	10
	Unclear subcutaneous tumours	4	4	2	12
	Soft tissue foreign bodies	4	4	2	12
	Evaluation of lymph nodes	1	8	1	10
	Abscess	5	4	1	14
Life threatening	Causes of cardiac arrest (4H, 4 T)	2	6	2	10
	FAST examination	7	2	1	16
	Verification of endotracheal tube placement	1	3	6	5

#### Barriers

The importance of barriers for using POCUS in family practice is presented in Table 2. Number of panellists for each barrier assessment is given. Different scores were assigned for different assessment: very important (2 points), important (1 point) or not important (0 points) and total sum of assigned points was calculated; *n* = 10.

**Table 2:** The importance of barriers for using POCUS in family practice

	Barriers	Very important (No. of panellists)	Important (No. of panellists)	Not important (No. of panellists)	Total score
Organisation	Not enough time for POCUS exam	7	3	0	17
	US device not available at all times	7	2	1	16
	Taking responsibility for POCUS exam	4	2	4	10
	Patients are not prepared for exam	0	6	4	6
	Not enough space for POCUS exam	0	5	5	5
	Negative opinion about POCUS	1	2	7	4
Education	Price of the courses	7	3	0	17
	Insufficient knowledge or experience	7	2	1	16
	Lack of specific education for FPs	7	2	1	16
	POCUS not used often enough	5	4	1	14
	Lack of tutors	5	4	1	14
Finance	High price of portable US device	6	4	0	16
	POCUS not paid by healthcare insurance	5	3	2	13

#### Knowledge and skills

The importance of required knowledge or skills for using POCUS in family practice is presented in Table 3. Number of panellists finding knowledge and skills very important (2 points), important (1 point) or not important (0 points) is given and total sum of assigned points was calculated; n = 10.

**Table 3:** The importance of required knowledge or skills for using POCUS in family practice

	Knowledge and skills	Very important (No. of panellists)	Important (No. of panellists)	Not important (No. of panellists)	Total score
Knowledge	Skills in clinical examination	10	0	0	20
	Clinical knowledge	10	0	0	20
	Knowledge of anatomy	10	0	0	20
	Knowledge of pathology	8	2	0	18
Skills	Handling of portable US device	9	1	0	19
	Knowledge of using and handling the probes	10	0	0	20
Education	Existing structured courses (e.g. Emergency POCUS course, Soft tissues US course etc.)	8	2	0	18
	Specific structured POCUS course for FPs	8	2	0	18
	Tutorship	9	1	0	19
	Possibility of one-to-one consultation with radiologist or experienced colleague	7	3	0	17
	Possibility of remote consultation with radiologist or experienced colleague	10	0	0	20

## Discussion

FPs are considering strengths and weaknesses of implementing POCUS into their everyday practice. The implementation of this technology is inevitably linked with the educational process of teaching and learning new knowledge and skills [13]. This study explored the indications and barriers for the use of POCUS among FPs in order to define a list of knowledge areas and skills to effectively implement POCUS in family practice.

A modified two-round Delphi method was used to conduct the study. Delphi method was used as a systematic opinion-gathering and decision-making technique [12]. The expert panel consisted of 13 panellists, ten (77%) completed both rounds of the study. In Slovenia, only a minority of FPs use POCUS in their everyday practice, therefore the target group was small. In informal enquiries about rather high rate of non-responders, some FPs expressed lack of confidence and experience in using POCUS.

The results of this study show a large variability of indications for the use of POCUS in family practice. FPs use POCUS for urgent and non-urgent situations, for dichotomous decision-making, screening, measurements of organs and structures and observation. The varied use of POCUS by FPs reflects a broad variety of work in family practice and in some cases decreased accessibility to other diagnostic methods [14, 15]. This finding is consistent with the European survey on variation in the use of POCUS [5], but indicates a wide variety of its use even within one country. The reasons for such variety can be due to special interests and working environments of FPs, as well as different initial trainings of POCUS. The strongest agreement on indications was the use of POCUS for acute abdominal conditions, followed by lung ultrasonography and eyeballing echocardiography. The visualisation of these structures needs fairly little training and experience; therefore they are included in a majority of initial POCUS trainings [8, 16]. Furthermore, a high incidence of positive findings in these indications makes a FP feel confident in using them and prioritise them highly on the indication list.

Some indications, such as cystitis, uterine myoma, ovarian cysts of confirmation of endotracheal tube placement, were often used by few panellists, but never used by a majority of panellists. This probably reflects specific needs of an individual FP and a wide diversity of work in family practice.

In contrast, the results of this study show little variability in barriers for the use of POCUS. The most common barriers are lack of time, inaccessibility of specific training programmes and financial issues. The barriers related to education are common to the majority of panellists. Similar observations were made in other studies [5, 10, 14, 15], although the majority of data in these studies refers to the use of POCUS in emergency settings, not in family practice. In order to enhance the use of POCUS in family practice, physicians should have more time per patient, a specific POCUS training for FPs, an affordable ultrasonography device available at all times and also financial incentives for POCUS examinations. However, the barriers should be further explored among the FPs, who are non-users of POCUS.

There is a strong consensus on the knowledge and skills a FP should have to perform POCUS. Panellists agreed on the broad content of medical knowledge, technical skills and educational options. They appreciate existing structured programmes, such as emergency POCUS courses and soft-tissue ultrasonography courses, but they recognise the need for a specific POCUS course for FPs. The content of the training programme should focus on specific medical knowledge (e.g. clinical examination, clinical skills, anatomy, and pathology) and technical skills (e.g. handling of a portable ultrasonography device and handling of probes). The most effective teaching methods for POCUS training programmes have been proven to be working in small groups, video-clips examples and hands-on sessions [17]. These methods are applicable also in specific POCUS training programmes for FPs.

Furthermore, panellists expressed a need for individual and remote consultations and tutorship options, during the formal educational process and beyond. Tutoring and a possibility of consultation are necessary to raise confidence and upgrade knowledge and skills to a level of competency [9].

This study has some limitations. First of all, the number of panellists is small and reflects a relatively low number of experts in Slovenia. The response rate of 42% could probably be improved by various incentives. However, we did not consider inclusion of other European countries in the study due to marked variability in the use of POCUS in Europe [5]. Secondly, the study was performed in 2016. POCUS technology is exposed to constant technology improvements and POCUS is becoming more and more affordable. This can reflect also in the attitudes of FPs towards the use of POCUS and their perception of barriers of POCUS use. Lastly, the study explored only conceptual knowledge and skills for performing POCUS. The exact content of an educational programme and its timeframe need yet to be defined.

Future work should focus on designing a specific POCUS training programme for established FPs both in vocational training and as continuous medical education. The programme should be specific for FPs, versatile in content, practical with hands-on approach, concise and in accordance with already existing courses. Moreover, the programme should include the possibility of individual tutoring and remote consultations.

## Conclusion

This study proves that although POCUS is used for a wide variety of indications with a significant number of barriers, there is a strong consensus on what a FP needs to know in order to effectively perform POCUS. A course of POCUS for established FPs should cover specific medical knowledge, practical skills in using POCUS device and varied learning possibilities with direct and remote tutorship and consultations. Designing such training programme is a further educational challenge for vocational and continuous medical education in family practice.

## Supplementary information

**Additional file 1.** Study questionnaire. Description of data: A two-round modified Delphi study questionnaire.

## Data Availability

All data generated or analysed during this study are included in this published article.

## Abbreviations

FP: Family physician
POCUS: Point-of-care ultrasonography
