# Supplementary material for: Knowledge and skills required to perform point-of-care ultrasonography in family practice – a modified Delphi study among family physicians in Slovenia
Source: BMC Fam Pract. 2020 Mar 26;21:56. doi: 10.1186/s12875-020-01130-z (PMC7098073; doi:10.1186/s12875-020-01130-z)
Supplement: Supplementary file 1 — Additional file 1. Study questionnaire. Description of data: A two-round modified Delphi study questionnaire. [file 12875_2020_1130_MOESM1_ESM.docx]

## Round-one questionnaire

**1. General information**

(1) Gender: male / female

(2) For how many years have you been working in family medicine?

(3) What is the setting of your family medicine practice? Urban / rural / mixed

(4) Do you provide home visits? Yes / no

(5) How often are you involved in the out-of-hours care or pre-hospital emergency service?

- I’m not involved in the out-of-hours care or pre-hospital emergency service
- I work mostly in the family practice and sometimes in the out-of-hours care or pre-hospital emergency service
- I work mostly in the out-of-hours care or pre-hospital emergency service and sometimes in the family practice
- I work only in the out-of-hours care or pre-hospital emergency service

**2. Study questions**

1. For which indications do you use point-of-care ultrasonography (POCUS) in your family practice?
2. What are the barriers of use of POCUS in a family practice?
3. What knowledge and skills does a family practitioner need to safely use POCUS in a family practice?

## Round-two questionnaire

**Question 1**

In the round-one questionnaire you have answered the questions about the indications of the use of POCUS in family practice. Your answers have been categorized, coded and grouped. Now we kindly ask you to indicate how often would you use POCUS in family practice for the following indications, if there were no limitations (e.g. time, financing…).

|  | Indication | Often | Sometimes | Never |
| --- | --- | --- | --- | --- |
| Lung evaluation | Pneumonia |  |  |  |
|  | Pleural effusion |  |  |  |
|  | Pneumothorax |  |  |  |
|  | Pulmonary embolism |  |  |  |
| Cardiovascular evaluation | Heart size and motility |  |  |  |
|  | Evaluation of hydration (vena cava compliance) |  |  |  |
|  | Pericardial effusion and heart tamponade |  |  |  |
|  | Abdominal aortic aneurism or dissection |  |  |  |
|  | Abdominal aortic aneurism (screening) |  |  |  |
|  | Deep venous thrombosis |  |  |  |
| Abdominal evaluation | Intra-abdominal free fluid |  |  |  |
|  | Ileus |  |  |  |
|  | Organomegaly |  |  |  |
|  | Gallbladder disease, gallstones |  |  |  |
|  | Hydronephrosis, renal stones |  |  |  |
|  | Retention of urine |  |  |  |
|  | Cystitis |  |  |  |
|  | Evaluation of prostate |  |  |  |
|  | Evaluation of testicles |  |  |  |
|  | Pregnancy (confirmation, evaluation) |  |  |  |
|  | Extra-uterine pregnancy |  |  |  |
|  | Uterine myoma |  |  |  |
|  | Ovarian cysts |  |  |  |
| Soft tissues evaluation | Injuries: muscle, tendon rupture, haematoma |  |  |  |
|  | Bursitis |  |  |  |
|  | Joint effusion |  |  |  |
|  | Fractured bone |  |  |  |
|  | Unclear subcutaneous tumours |  |  |  |
|  | Soft tissue foreign bodies |  |  |  |
|  | Evaluation of lymph nodes |  |  |  |
|  | Abscess |  |  |  |
| Emergency | Causes of cardiac arrest (4H, 4T) |  |  |  |
|  | FAST examination |  |  |  |
|  | Verification of endotracheal tube placement |  |  |  |

**Question 2**

In the round-one questionnaire you have answered the questions about the barriers for the use of POCUS in family practice. Your answers have been categorized, coded and grouped. Please, indicate how important do you find the barriers for the use of POCUS in family practice.

|  | Barrier | Very important | Important | Not important |
| --- | --- | --- | --- | --- |
| Organisation | Not enough time for POCUS exam |  |  |  |
|  | US device not available at all times |  |  |  |
|  | Taking responsibility for POCUS exam |  |  |  |
|  | Patients are not prepared for exam |  |  |  |
|  | Not enough space for POCUS exam |  |  |  |
|  | Negative opinion about POCUS |  |  |  |
| Education | Price of the courses |  |  |  |
|  | Insufficient knowledge or experience |  |  |  |
|  | Lack of specific education for FPs |  |  |  |
|  | POCUS not used often enough |  |  |  |
|  | Lack of tutors |  |  |  |
| Finance | High price of portable US device |  |  |  |
|  | POCUS not paid by healthcare insurance |  |  |  |

**Question 3**

In the round-one questionnaire you have answered the questions about the required knowledge and skills for the use POCUS in family practice. Your answers have been categorized, coded and grouped. Please, indicate how important do you find the listed knowledge and skills for the use POCUS in family practice.

|  | Knowledge and skills | Very important | Important | Not important |
| --- | --- | --- | --- | --- |
| Knowledge | Skills in clinical examination |  |  |  |
|  | Clinical knowledge |  |  |  |
|  | Knowledge of anatomy |  |  |  |
|  | Knowledge of pathology |  |  |  |
| Skills | Handling of portable US device |  |  |  |
|  | Knowledge of using and handling  the probes |  |  |  |
| Education | Existing structured courses (e.g. Emergency POCUS course, Soft tissues US course etc.) |  |  |  |
|  | Specific structured POCUS course for FPs |  |  |  |
|  | Tutorship |  |  |  |
|  | Possibility of one-to-one consultation with radiologist or experienced colleague |  |  |  |
|  | Possibility of remote consultation with radiologist or experienced colleague |  |  |  |
